# Acute Effect of Different Resisted Sprint Loads on Joint Kinematics and Electromyographic Activity in Experienced Sprinters

**DOI:** 10.1155/tsm2/2139446

**Published:** 2026-10-14

**Authors:** Roland van den Tillaar, Sam Gleadhill, Pedro Jiménez-Reyes, Ryu Nagahara

**Affiliations:** ^1^ Department for Sports Science and Physical Education Nord University Levanger Norway nord.no; ^2^ College of Health Adelaide University Adelaide Australia adelaide.edu.au; ^3^ Sports Sciences Research Centre Rey Juan Carlos University Madrid Spain urjc.es; ^4^ Faculty of Sports and Budo Coaching Studies National Institute of Fitness and Sports in Kanoya Kanoya Japan nifs-k.ac.jp

**Keywords:** angles, electromyography, resisted sprinting, segmental angular velocity, velocity decrement

## Abstract

**Purpose:**

The aim of this study was to investigate the acute effect of different active resisted sprint loads upon joint kinematics and leg electromyography (EMG) activity in experienced sprinters.

**Methods:**

Thirteen experienced male sprinters (age: 20.5 ± 1.3 years, body mass: 66.8 ± 4.5 kg and body height: 1.74 ± 0.06 m) with best 100‐m times of 11.24 ± 0.34 s performed a 50‐m sprint with maximal effort from three‐point start. Afterwards, they performed different resisted sprints with maximal effort provided by a robotic pulley system that resulted in 65%, 50%, 25% and 10% reduction of peak velocity for, respectively, 10, 20 and 30 m in randomised order while spatiotemporal parameters, joint kinematics and EMG of nine leg muscles were measured from the point which sprint velocity plateaued.

**Results:**

The main findings were that increasing velocity‐decrement conditions resulted in reduced sprint velocity, shorter step length, lower step frequency and prolonged ground contact time together with joint kinematics changing to a more flexed posture and forward leaning at touch down and less extension at toe off, with lower, maximal angular velocities during contact times. These adaptations resulted in more quadriceps dominant activity with increased stabilizing activity of gluteus medius, while hamstring activity decreased due to the lower angular knee extension during flight times with increasing resisted loads.

**Conclusion:**

This study suggests that while resisted sprinting is an effective tool for increasing force production demands, higher resisted loads (> 50% velocity decrement) are associated with progressively different kinematic and neuromuscular patterns, likely reflecting the combined effects of increased force demands and thereby reduced running velocity.

## 1. Introduction

Sprint performance is a critical component in many sports, like athletics, football, hockey and other team‐based activities, where the ability to accelerate rapidly over short distances often determines success in decisive moments. Acceleration performance, particularly within the first 10–40 m, has therefore been widely studied, with research highlighting the importance of force production, technique and neuromuscular coordination in achieving high sprint velocities.

One commonly applied method to enhance sprint acceleration is resisted sprint training, typically performed using sled towing, parachutes or motorized resistance devices [1–3]. This approach is designed to increase the mechanical demands placed on the athlete, particularly in terms of horizontal force production. Alcaraz et al. [4] indicated in their systematic review that it can improve acceleration performance, especially in the early phase of sprinting. However, Aldrich [5] in a more recent meta‐analyses has suggested that the improvements observed with resisted sprinting are generally small and may not be superior to unresisted sprint training when training volume is matched. In addition, studies investigating different loading conditions have reported that heavier loads may enhance force production capabilities (e.g., increases in theoretical maximal force), whereas lighter loads may be more specific to sprint velocity development [6]. Despite these findings, there remains considerable variability in the literature regarding optimal loading strategies, likely due to differences in methodology, participant characteristics and load prescription approaches [7].

Most existing studies on resisted sprinting have primarily focussed on spatiotemporal parameters, such as sprint time, stride length, stride frequency and contact time [8–11]. These investigations consistently demonstrate that increasing resistance reduces sprint velocity, shortens stride length and increases contact time. While such measures provide valuable performance‐related information, they offer limited insight into the underlying biomechanical and neuromuscular mechanisms responsible for these changes. In contrast, relatively few studies have examined joint kinematics during resisted sprinting [1, 11, 12], and even fewer have incorporated electromyography (EMG) to assess muscle activation patterns [13, 14]. The limited available evidence suggests that resisted sprinting can alter running mechanics, including increased trunk lean and modified joint angles, particularly under heavier loads [11, 15]. However, these findings are not consistent across studies, and the lack of integrated analyses combining kinematics and EMG makes it difficult to fully understand how movement strategies and neuromuscular control are affected.

Furthermore, most EMG‐based studies have investigated only a restricted range of loading conditions, typically comparing a small number of discrete loads (e.g., 5%–15% body mass), rather than examining responses across the full load‐velocity spectrum. For example, recent work examining neuromuscular responses to resisted sprinting reported minimal changes in muscle activation across different loads, despite clear decrements in sprint performance [16]. This suggests that the relationship between external load and neuromuscular adaptation remains unclear and warrants further investigation.

Therefore, the aim of this study was to investigate the acute effects of a wide range of active resisted loads across the full load spectrum on joint kinematics and EMG activity during sprinting in experienced sprinters. By integrating biomechanical and neuromuscular analyses, this study seeks to provide a more comprehensive understanding of how resisted sprinting influences movement strategies and muscle activation patterns.

## 2. Methods

Thirteen experienced male sprinters (age: 20.5 ± 1.3 years, body mass: 66.8 ± 4.5 kg and body height: 1.74 ± 0.06 m) with best 100‐m times of 11.24 ± 0.34 s were recruited from the local athletics club. Informed consent was obtained from each participant prior to the study. The study complied with current ethical regulations for research and was approved by the institutional research ethics committee (#11–54 on 14^th^ of September 2018) according to the guidelines of the Declaration of Helsinki. A sample size determination was based on a repeated measures design upon maximal sprint velocities during pilot testing with the different resisted loads with an *α* = 0.05, power = 0.80, Δ = 1.2 m/s and SD = 0.25 m/s. A minimal sample size of only 4 athletes was required.

The sprinters performed three sessions: two familiarisation sessions to establish the different decreases in velocities (10%, 25%, 50% and 65%) by sprinting with active resistance provided by a robotic pulley system (DynaSpeed, Ergotest Innovation AS, Stathelle, Norway) and one test session with at least two days between each session. The testing session was performed on an indoor athletic track. After a self‐selected warm up (in general consisting of jogging, dynamic exercise to increase range of motion and some short sprints at submaximal level) under supervision of the researchers, participants performed one–two unresisted 50‐m sprints with maximal effort from three‐point start, wearing spikes. This was followed by a 10, 20 and 30‐m resisted sprints with maximal effort with an active resistance that caused a, respectively, 65%, 50%, 25% and 10% reduction of peak velocity in a randomised order counterbalanced across sprinters with 5‐6‐min rest between each sprint. Shorter distances were covered with increasing active load to avoid decreasing velocity at end of distance due to possible fatigue as found by pilot testing. Before the test session, the participant put on the Xsens lycra suit (Xsens Technologies B.V. Enschede, Netherlands), which consisted of 17 IMUs (240 Hz) attached to the athletes’ spikes, shanks, thighs, pelvis, sternum, head, upper arms and forearms. After calibration (posing in N‐pose and walking around), the software Xsens MVN v. 2024.2 (Movella, Enschede, Netherlands) automatically calculated joint angles and joint velocities over time. Sensor fusion for IMU data was performed using the patented algorithms (Xsens Kalman Filter) and filtered using the LXsolver (minimize soft tissue artefact) in MVN Biomech Studio. This system has shown to be good to excellent reliability and waveform agreement in the sagittal plane during high‐speed running and sprinting, with high intraclass correlation coefficients (ICCs > 0.88–0.90) for hip, knee and ankle flexion‐extension angles [17, 18]. For each step at touch down and toe off, which was based upon the algorithm of van den Tillaar et al. [19] in each sprint and checked by visual inspection and software (MVN Analyze 2024.2, Xsens, Netherlands), joint angles in the sagittal plane (ankle, knee and hip) were identified and used for further analysis. Furthermore, minimal and maximal joint angles (ankle, knee and hip, Figure 1) and maximal angular joint velocities (ankle, knee and hip) in sagittal plane during the contact and flight phase in each step were recorded. The reliability of the kinematic measurements in the sagittal plane was found to be with ICC ≥ 0.92 as found in a previous study [17].

**FIGURE 1:**
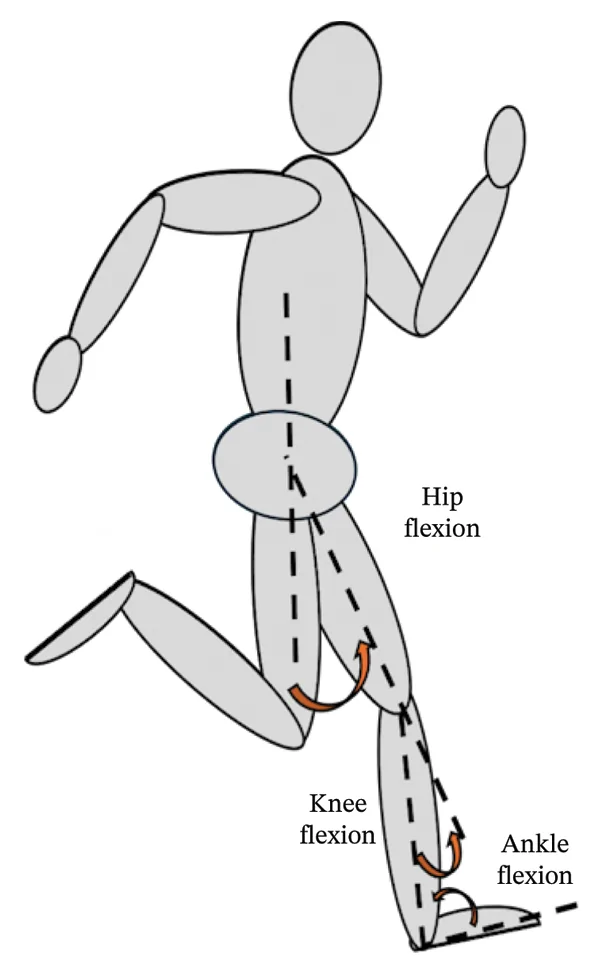
Definition of joint angles of hip, knee and ankle flexion.

From each sprint, the mean spatiotemporal and joint kinematics were calculated from the point at which the participant’s sprint velocity plateaued out, also called the second breakpoint or transition point [20, 21] until the end of the measured distance, hereafter referred to as the condition‐specific maximal velocity phase. Furthermore, the step and distance at which this was reached were recorded.

Distance over time measurements were recorded continuously during each attempt using a CMP3 Distance Sensor laser gun (Noptel Oy, Oulu, Finland), sampling at 2.56 KHz. Velocity was automatically calculated as part of the MUSCLELAB system (Ergotest Technology AS, Stathelle, Norway). Two wireless, 9 degrees of freedom, IMUs integrated with a 3‐axis gyroscope (sampling rate of 1000 Hz) were attached on top of the shoelaces of the spikes of each foot to identify contact and flight times automatically by the software together with step length (distance between two adjacent contact times measured with laser) and frequency (1/(contact + flight times)) as the laser was synchronized with the MUSCLELAB software v. 10.251 (Ergotest Technology AS, Langesund, Norway), which had very high correlations (*r* > 0.92) with a 50‐m force plate system [19]. The IMUs were fixed by tape over the spikes exactly under the IMUs of the Xsens system, which allowed both systems to identify touchdown and toe‐off events (abrupt dip in angular velocity and increase in acceleration of IMU) [22] synchronically as both IMUs showed the same signals which could recognise these events [19]. Contact and flight time of the right leg was used for further analysis as EMG was attached to the right leg in which EMG was averaged for a flight and contact phase, while the other spatiotemporal and joint kinematics were analysed for each step as no significant differences between left and right were observed during the maximal velocity phases of each decrement‐velocity condition.

EMG activity of the muscles was measured by using a wireless unit with a sampling rate of 1 kHz (Ergotest Innovation, Porsgrunn, Norway) with electrodes Dri‐Stick Silver circular sEMG Electrodes AE‐131 (NeuroDyne Medical, Germantown, TN, USA) on the muscles of the right leg. The skin to which the electrodes were fastened had been shaved and washed with alcohol before fastening the electrodes. The electrodes (11‐mm contact diameter and fixed 2‐cm centre‐to‐centre distance) were placed along the presumed direction of the underlying muscle fibres on the lateral and medial vastii, rectus femoris, biceps femoris, semitendinosus, soleus, lateral gastrocnemius, gluteus medius and gluteus maximus muscles according to the recommendations of SENIAM by the same researcher for all athletes [23]. The EMG raw signal was amplified by 400 and filtered using a preamplifier located as close as possible to the pickup point with the intention of minimising the noise induced from external sources through the signal cables. The preamplifier had a common mode rejection ratio of 100 dB. The EMG raw signal was then bandpass filtered (fourth‐order Butterworth filter) with cut‐off frequencies of 20 Hz and 500 Hz. The resulting EMG signals were converted to moving root mean square (RMS) signals with a smoothing time window of 10 ms for the contact and flight phases of each step on right side. All sensors were synchronised using MUSCLELAB Version 10.251 (Ergotest Innovation, Porsgrunn, Norway), which made it possible to measure and analyse kinematics and muscle activity for each stride cycle. To compare EMG activity between the conditions, EMG normalisation was performed by using the highest mean RMS of a phase for each muscle as a normalisation signal for each participant. This has shown to be a reliable, repeatable and sensitive method (ICC > 0.80) like the MVC method for normalising and measuring EMG in sprinting and is suggested to be the most appropriate for EMG normalisation by showing better intra‐subject reliability, repeatability and better sensitivity during sprinting over different days and for once‐off measurements than the MVC method [24, 25].

### 2.1. Statistical Analysis

To check for normality, the Shapiro–Wilk test of normality was used and confirmed. To investigate similarities and differences in joint kinematics and EMG activity between the different maximal velocity phases of each active resisted load, a one‐way ANOVA with repeated measurements was performed on the spatiotemporal parameters and joint kinematics, while a two‐way (load and phase: contact and flight) repeated‐measures ANOVA on EMG activity was used. When significant differences were observed, post hoc comparisons with Holm–Bonferroni correction were performed to avoid the risk of making Type I errors. Effect size was evaluated with partial eta squared where 0.01 < $\eta_{p}^{2}$ < 0.06 constitutes a small effect, 0.06 < $\eta_{p}^{2}$ < 0.14 a medium one and $\eta_{p}^{2}$ > 0.14 a large effect [26]. Where the sphericity assumption was violated, the Greenhouse–Geisser adjustments of the *p* values were reported. The level of significance was set at *p* < 0.05. All data analyses were performed using JASP v. 0.19.1 (University of Amsterdam, Amsterdam, Netherlands). Data were presented as means and 95% confidence intervals (95% CIs).

## 3. Results

The active resistance used increased from 3.5 to 32.6 kg, which resulted in velocity reductions that were targeted, except for the 65% reduction. This reduction was only 60.1%. The start of the condition‐specific maximal velocity plateau decreased from 9.9 to 4.1 m with decreased step number, and the steps used for analysis were also decreased from 21.5 to 7.3 steps (Table 1). In general, most sprinters performed one extra sprint over all conditions as problems with measurements occurred on average one trial per sprinter per session. Thus, a total of six sprints (10–50 m) with maximal effort were performed per sprinter.

**TABLE 1:** Average (±95% CI) load, actual velocity decrement, sprint distance, start plateau and number of steps per condition.

	Unresisted	10%	25%	50%	65%
Active resistance (kg)	—	3.5 ± 0.7	11.0 ± 0.5	24.3 ± 1.1	32.6 ± 1.6
Actual velocity reduction (%)	0	11.1 ± 0.7	25.9 ± 1.0	49.3 ± 0.7	60.1 ± 1.3
Sprint distance (m)	50	30	30	20	10
Start plateau (step)	7.8 ± 0.3	6.6 ± 0.4	6.4 ± 0.04	4.7 ± 0.5	4.5 ± 0.3
Start plateau (m)	9.9 ± 0.5	9.2 ± 0.7	7.2 ± 0.6	4.7 ± 0.5	4.1 ± 0.4
Used steps (number)	21.5 ± 0.6	12.8 ± 0.9	15.9 ± 1.0	14.8 ± 0.8	7.3 ± 0.6

### 3.1. Spatiotemporal Parameters

Condition‐specific maximal velocity and all other spatiotemporal parameters were significantly affected by the velocity‐decrement conditions (F_(4,48)_ ≥ 8.8, *p* < 0.001, $\eta_{p}^{2}$ ≥ 0.40). Post hoc comparisons revealed that maximal velocity, step length and frequency decreased, while contact times increased with each velocity‐decrement condition. In addition, the only significant decrease in flight time was observed from 25% to 50% velocity‐decrement conditions (Figure 2).

**FIGURE 2:**
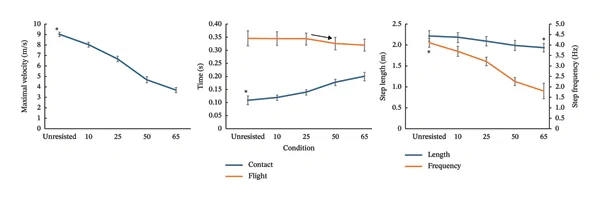
Mean ± 95% CI velocity and spatiotemporal parameters for unresisted and different resisted loads. ^∗^Indicates a significant difference between all conditions. ⟶ indicates a significant change between these two loads and all right from the arrow.

### 3.2. Joint Kinematics

All joint angles at touch down, toe off and maximal angles were significantly affected by the velocity‐decrement condition (F_(4,48)_ ≥ 3.82, *p* < 0.01, $\eta_{p}^{2}$ ≥ 0.27). Post hoc comparison revealed that touch down hip and knee flexion angles increased with each velocity‐decrement condition, while dorsal flexion angle only significantly increased from 10 to 25 and from 25% to 50% velocity‐decrement condition. At toe off, hip extension decreased with each load from 10% to 65%, while knee flexion decreased only significantly from 25% to 50% velocity‐decrement condition. Plantar flexion angle was only significantly less with the highest velocity‐decrement condition of 65% compared to all other loads (Figure 3). The maximal hip flexion during the flight phase increased, while maximal knee flexion during the flight phase decreased with each load. Maximal dorsal flexion angle increased significantly with each load except not from 50% to 65% (Figure 4).

**FIGURE 3:**
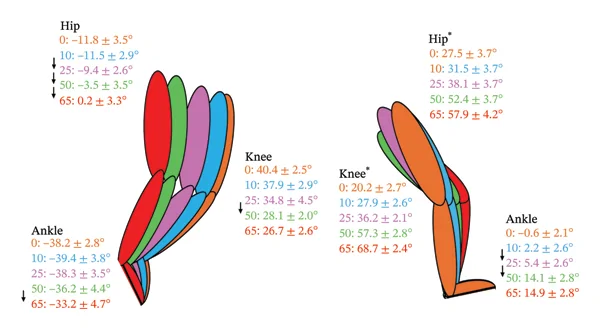
Mean ± 95% CI joint angles at touch down and toe off for unresisted and different resisted loads. ^∗^Indicates a significant difference between all velocity‐decrement conditions. ⟶ indicates a significant change between these two loads and all down for it.

**FIGURE 4:**
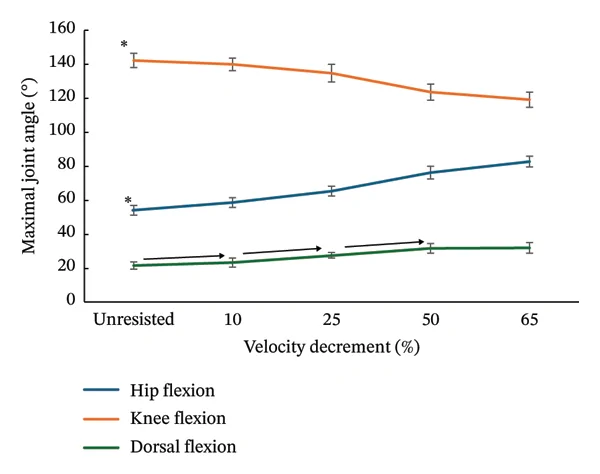
Mean ± 95% CI peak hip flexion and knee flexion during the flight phase and peak dorsal flexion during the contact phase for unresisted and different resisted loads. ^∗^Indicates a significant difference between all velocity‐decrement conditions. ⟶ indicates a significant change between these two loads and all right of the arrow.

Also, all the peak angular joint velocities during flight and contact phases were significantly affected by the velocity‐decrement conditions (F_(4,48)_ ≥ 5.00, *p* < 0.002, $\eta_{p}^{2}$ ≥ 0.33). Post hoc comparison revealed that peak hip extension and dorsal and plantar flexion velocities during the contact phase significantly decreased from 10 to 25 and from 25% to 50% velocity‐decrement conditions, while during the flight phase, hip flexion velocity increased from unresisted to 10 and from 10% to 25% velocity‐decrement conditions and peak hip extension velocity significantly increased from 50% to 65% velocity‐decrement condition. Peak knee flexion velocity during the flight phase and knee extension velocity during the contact phase decreased with each velocity‐decrement condition, while peak knee extension velocity during the flight phase only significantly increased from 10% to 25% velocity‐decrement condition (Figure 5).

**FIGURE 5:**
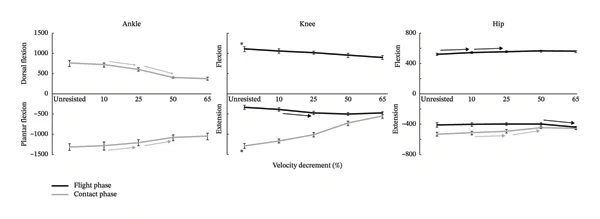
Mean ± 95% CI peak angular joint velocity during the contact and flight phase for unresisted and different resisted loads. ^∗^Indicates a significant difference between all loads. ⟶ indicates a significant change between these two loads and all right of the arrow.

### 3.3. EMG

A significant effect was found for velocity decrement for the gluteus medius, gastrocnemius, rectus femoris and the two hamstring muscles (F_(4,48)_ ≥ 3.19, *p* < 0.031, $\eta_{p}^{2}$ ≥ 0.35), while a significant effect between the contact and flight phase was found for the gluteus maximus, soleus, gastrocnemius, medial and lateral vastii and semitendinosus (F_(4,48)_ ≥ 6.44, *p* < 0.044, $\eta_{p}^{2}$ ≥ 0.52). In addition, a significant interaction effect was found for all three quadriceps muscles (F_(4,48)_ ≥ 4.23, *p* < 0.008, $\eta_{p}^{2}$ ≥ 0.38). Post hoc analysis revealed that the hamstring muscles decreased EMG activity with velocity‐decrement condition, especially between the 25% and 50% velocity‐decrement conditions for both flight and contact phases. Gluteus medius increased its EMG activity significantly from unresisted to 25% and 65% decrement loads for, respectively, the contact and flight phases, while for the gastrocnemius, only EMG activity decreased significantly for the flight phase from 25% to 50% decrement. For the rectus femoris and medial vastus, the activity increased only in the contact phase significantly from 25% with the higher velocity‐decrement conditions, while in the flight phase, no significant changes were observed resulting in a significant interaction effect. Also, a significant interaction effect was found for the lateral vastus by an increase in EMG activity during the contact phase while flight phase EMG activity was relatively constant (Figure 6).

**FIGURE 6:**
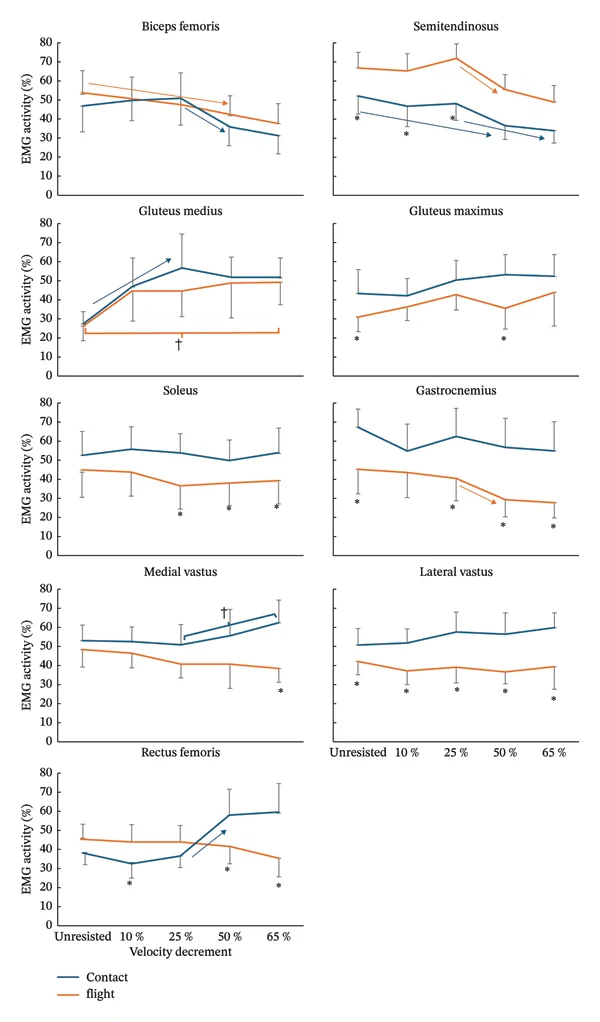
Mean ± 95% CI for all muscles per condition and contact and flight phase. ^∗^Indicates a significant difference between the two phases for this condition. ^†^Indicates a significant difference between these two conditions for this phase. ⟶ indicates a significant change between these two loads for this condition and all right of the arrow.

EMG activity was significantly higher in the contact phase for both plantar flexor muscles, gluteus maximus and the three quadriceps muscles than activity during the flight phase, while the semitendinosus showed the opposite. However, while EMG activity during the contact phase was significantly higher for almost all load decrements for the gastrocnemius and lateral vastus, for the rectus femoris, soleus and medial vastus, the difference in EMG was significantly different with the higher velocity‐decrement conditions (Figure 6). For the semitendinosus, the difference between the phases was only significant with the unresisted and lower two velocity‐decrement conditions, while for the gluteus maximus, only the unresisted and 50% velocity‐decrement conditions reached significance level (Figure 6).

## 4. Discussion

The present study investigated the effects of progressive active velocity‐decrement conditions on sprint kinematics and neuromuscular activity, revealing substantial alterations in spatiotemporal parameters, joint kinematics and muscle activation patterns. Overall, increasing velocity‐decrement conditions resulted in reduced sprint velocity, shorter step length, lower step frequency and prolonged ground contact time. These findings are consistent with previous resisted sprint studies, which have consistently reported that increasing external load modifies sprint mechanics primarily through reductions in running velocity and increased force requirements [7, 27]. The progressive decrease in step length and frequency alongside increased contact time likely reflects a shift towards a more force‐oriented, rather than velocity‐oriented, sprinting strategy under higher loads. Importantly, as sprint mechanics are strongly velocity‐dependent, the present findings should be interpreted within the context of the reduced running velocities induced by each resisted condition.

The velocity decrement of 65% was actually 60.1%, which was significantly less than expected based upon the two familiarisation sessions. This discrepancy can be explained by the fact that the sprinters did not have experience with such an active resisted load during sprinting before. Furthermore, the sprinters could be extra excited during the test session, which could result in higher velocities with this load.

While most spatiotemporal parameters changed with each velocity‐decrement condition, flight time only significantly reduced between the 25% and 50% velocity‐decrement conditions, suggesting a potential threshold beyond which flight phase mechanics are more substantially altered. This was in accordance with previous studies that showed that at condition‐specific maximal velocity, not much change in flight times occurs when resisted loads are small [28, 29], while with larger active resisted loads, flight times decrease [10].

The decreased sprint velocity and changed spatiotemporal parameters were also clearly visible in joint kinematics. The observed increases in hip, knee and dorsal ankle flexion at touchdown with increasing load indicate a more crouched limb posture during ground contact (Figure 3). This adjustment has been previously described as a compensatory strategy to enhance force production duration when resistance increases [14, 15]. Thereby, the centre of mass is lower, with more forward lean to have more body mass pulling to resist the load. In so doing, at toe off, the progressive reduction in hip extension and knee flexion indicates a diminished ability to achieve full extension, due to the more forward lean. This likely contributes to the reduced step length observed. The reduction in plantarflexion at the highest load (65%) further suggests a limitation in propulsive capacity at extreme resistances. Furthermore, during the flight phase, increased hip flexion and reduced knee flexion indicate a modified recovery strategy, while the ankle dorsal flexion angle increases during the contact phase (Figure 4). This may reflect an attempt to reposition the limb more rapidly under constrained velocity conditions and to overcome the active resistance [15]. This repositioning of joint angles with increasing load was clearly the result of reductions in peak angular velocities at the hip, knee and ankle during the contact phase. This highlighted a general slowing of limb movement under resistance. This supports previous findings that resisted sprinting reduces segmental velocities due to increased force demands [30]. In contrast, the increase in hip flexion velocity during the flight phase at lower loads (up to 25%) and increased hip extension velocity at heavy loads (Figure 5) suggest a compensatory strategy of repositioning to keep the athlete in the position to propel the body forward as best as possible.

Due to the repositioning of the body with increasing resistance, muscle activity had to adapt. Similarly, the quadriceps muscles (rectus femoris and vasti) showed increased activation during the contact phase at higher loads, indicating a shift towards greater knee extensor contribution which is due to the more forward lean. The interaction effects observed between phases further highlight the phase‐specific nature of neuromuscular adaptations, with contact phase demands becoming increasingly dominant under higher loads. This was in line with Zabaloy et al. [14] who also showed that with heavy loads, quadriceps activity increased, while Bartosz‐Jeffries et al. [16] showed that with low loads (15% body mass), no difference in quadriceps activity was observed. Increased activation of the gluteus medius suggests a greater requirement for pelvic stabilization as load increases [31, 32]. The decreased hamstring activity with increasing load is expected as the hamstrings are mainly active during the late swing phase to decelerate knee extension [33, 34]. As knee extension velocity decreases with increasing velocity‐decrement conditions (Figure 5), hamstring activity decreases. The greater EMG activity in the contact phase for most muscles aligns with the fundamental role of ground contact in force production during sprinting [35]. The exception observed in the semitendinosus showed higher activation during the flight phase at lower loads. This reflects its role in late swing phase deceleration of the limb at high velocity [33, 34]. However, no kinetic analysis was performed in this study that could confirm the statements about kinematics and EMG activity in relation to force production. Thus, this should be investigated in future studies to confirm if there is a direct relationship between the kinematic and EMG changes with changes in force production.

Even with these interesting findings, the study has some limitations. Only a few athletes were tested from a certain level in a single session. Thereby, it can be different for female athletes, youth athletes, team‐sport populations or other level of sprinters, especially for elite sprinters as they in general are stronger and have possible higher kinematic outcomes resulting in faster times. Sprint distances varying from 50 to 10 m were used with the different resisted loads, which resulted in different steps used (21.5–7.3) for the analysis, which could influence the findings. However, using the same distance for all conditions would certainly introduce fatigue (decrease of velocity over longer distance) with the higher resisted loads, which was visible during pilot testing. Therefore, these distances were used to avoid notable decrease of sprint velocity during the resisted sprints. In addition, active pulling resistance of different levels was used, which is different from using sled towing, parachutes and weighted vests due to the active pull, also when standing still. This probably resulted in higher efforts during the sprint compared with similar weighted sleds and vests. This makes it difficult to make a direct comparison on spatiotemporal joint kinematics and EMG with these types of resisted sprinting, which should be investigated in future studies. Furthermore, there was a lot of variability in EMG measurements between the different athletes, which makes it difficult to compare all measurements with each other. Furthermore, kinematics and EMG measurements were only performed unilaterally and analysed for flight and contact phases, which does not give us a full insight of the sprint strategies with different velocity‐decrement conditions. Also, no kinetic measures were performed to relate the EMG measurements to the force output during the different conditions. Another limitation of the present study is that the addition of resistance resulted in a reduction in movement velocity. Therefore, it is difficult to distinguish whether the observed changes in kinematics and muscle activation were caused by the resistance itself or by the accompanying decrease in movement velocity. Therefore, future studies should investigate longitudinal changes in sprint performance in relation to changes in kinematics, kinetics and EMG with the different velocity‐decrement conditions and velocity‐matched unresisted conditions with elite level athletes to gain more insight over what causes the changes and possible mechanisms behind it for each athlete.

## 5. Conclusion

Based upon the findings of the study, it was concluded that active resisted sprints with maximal effort over a large range at condition‐specific maximal velocity affect spatiotemporal and joint kinematics and muscle activity by slower maximal speed, longer contact time and shorter step and flight times, while joint kinematics indicate changes to a more crouched position and forward leaning at touch down and less extension at toe off, with lower, maximal angular velocities during contact times due to the increasing load for this particular level of athletes using this specific active robotic pulley system. These adaptations resulted in more quadriceps dominant activity with more stabilizing activity of the gluteus medius, while hamstring activity decreases due to the lower angular knee extension during flight times with increasing resisted loads. This study suggests that resisted sprinting is an effective tool for increasing force production demands, indicating that increasing resisted loads progressively modify sprint kinematics and neuromuscular coordination, reflecting a shift towards more force‐oriented movement strategies under reduced running velocities. However, due to the low number of athletes of a certain level, these outcomes should be seen as exploratory with maintaining appropriate caution. However, because of the limited number at a given performance level, these findings should be regarded as exploratory and interpreted cautiously. From an applied perspective, different loading conditions should be viewed as targeting distinct regions of the sprint load–velocity spectrum rather than representing optimal or suboptimal techniques [36]. Therefore, practitioners should carefully prescribe resisted sprint loads depending on training goals. Lighter loads may better preserve sprint mechanics at higher velocities, whereas heavier loads may be more suited for a potential force‐oriented stimuli, depending on the specific training objective.

## Funding

This research received no specific grant or funding from any funding agency in the public, commercial or not‐for‐profit sectors.

## Conflicts of Interest

The authors declare no conflicts of interest.

## Data Availability

The data presented in this study are available upon request from the corresponding author. The data are not publicly available due to the national laws of the Norwegian government regarding privacy.
